# Thermal Side-Channel Threats in Densely Integrated Microarchitectures: A Comprehensive Review for Cyber–Physical System Security

**DOI:** 10.3390/mi16101152

**Published:** 2025-10-11

**Authors:** Amrou Zyad Benelhaouare, Idir Mellal, Michel Saydé, Gabriela Nicolescu, Ahmed Lakhssassi

**Affiliations:** 1 Department of Engineering and Computer Science, University of Quebec in Outaouais, Gatineau, QC J9A 1L8, Canada; idir.mellal@gmail.com (I.M.); michel.sayde@uqo.ca (M.S.); ahmed.lakhssassi@uqo.ca (A.L.); 2 Department of Computer Engineering and Software Engineering, Polytechnique Montreal, Montreal, QC H3C 3A7, Canada; gabriela.nicolescu@polymtl.ca

**Keywords:** densely integrated microarchitectures, 3D-ICs, SiP, chiplet-based design, TSCAs, CPS, OT, cybersecurity, thermal leakage, advanced microelectronics, RAII

## Abstract

Densely integrated microarchitectures spanning three-dimensional integrated circuits (3D-ICs), chiplet-based designs, and system-in-package (SiP) assemblies make heat a first-order security concern rather than a mere reliability issue. This review consolidates the landscape of thermal side-channel attacks (TSCAs) on densely integrated microarchitectures: we systematize observation vectors and threat models, clarify core concepts and assumptions, compare the most credible evidence from the past decade, and distill the main classes of defenses across the hardware–software stack. We also explain why hardening against thermal leakage is integral to cyber–physical system (CPS) security and outline the most promising research directions for the field. The strategic relevance of this agenda is reflected in current policy and funding momentum, including initiatives by the United States Department of Homeland Security and the Cybersecurity and Infrastructure Security Agency (DHS/CISA) on operational technology (OT) security, programs by the National Science Foundation (NSF) on CPS, and Canada’s Regional Artificial Intelligence Initiative and Cyber-Physical Resilience Program (RAII, >CAD 35 million), to bridge advanced microelectronics with next-generation cybersecurity. This survey offers a clear, high-level map of the problem space and a focused baseline for future work.

## 1. Introduction

### 1.1. General Context of Miniaturization and Heterogeneous Integration

Since the advent of integrated circuits, microelectronics has followed a sustained trajectory of miniaturization driven by Moore’s Law, which predicts a doubling of the number of transistors every 18 to 24 months. This trend has led to a significant reduction in technology nodes, from 3 micrometers (µm) in 1987 to 3 nanometers (nm) in 2022, resulting in substantial gains in performance and energy efficiency. For example, TSMC, one of the world’s leading semiconductor foundries, successively delivered process technologies at 7 nm (2018), 5 nm (2020), and 3 nm (2022), each providing approximately 10–15% improvement in performance, 25–30% reduction in power consumption, and a 30% increase in logic density compared to the previous node [1,2].

This progression has also been mirrored by Samsung and Intel, which have adopted these advanced nodes in their latest processor families, confirming the ongoing pursuit of miniaturization, although it is increasingly constrained by physical phenomena such as quantum tunneling and the reliability limitations of transistors at atomic scales [3,4,5].

**Figure 1:**
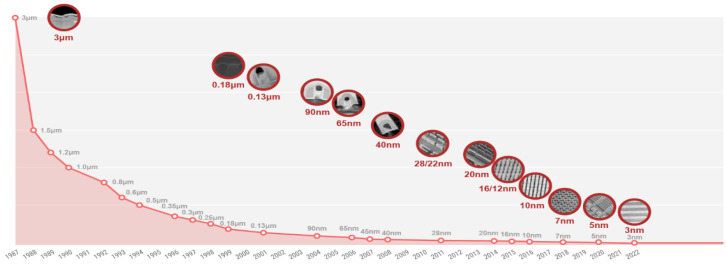
Historical evolution of semiconductor process nodes from 3 µm to 3 nm in TSMC technologies (1987–2022) [1].

Figure 1 presents a chronological overview of the evolution of TSMC’s major technology nodes from 1987 to 2022, highlighting the continuous shrinkage of critical dimensions and the associated gains in performance and density.

After several decades of innovation driven by Moore’s Law and later by the “More-than-Moore” paradigm, the semiconductor industry has not halted its pursuit of performance through miniaturization. In response to the exponential growth in computational demands and data-intensive applications, a new trend has emerged with the rise of Huang’s Law. Formulated by Jensen Huang, CEO of NVIDIA, this principle suggests that the performance of Graphics Processing Units (GPUs), particularly their parallel processing capabilities, is increasing at a rate significantly faster than what transistor scaling alone would allow. According to Huang’s Law, GPUs’ computational power doubles approximately every two years, propelled by a combination of advances in hardware architecture, software optimization, and unprecedented levels of parallelism enabled by these processors [6].

This unprecedented acceleration in computing has led to the development and widespread adoption of advanced integration technologies such as Very-Large-Scale Integration (VLSI) and Ultra-Large-Scale Integration (ULSI) circuits, multiprocessor systems on chip (MPSoCs) [7], three-dimensional integrated circuits (3D-ICs) [8], chiplet-based architectures [9], and system-in-package (SiP) solutions [10]. These innovations address the growing demand for massive parallel processing by maximizing the synergy between heterogeneous processing units (CPU, GPU, AI accelerators) [11]. Whether for scientific computing, modeling, imaging, or artificial intelligence, these large-scale integrated architectures are redefining the very concept of embedded systems and modern data centers by delivering unprecedented processing density and functionality.

### 1.2. Persistent Thermal Management Challenges

However, this upward trajectory in integration and performance inevitably brings forth critical thermal challenges. As systems become denser and more functionally compact, the ability to effectively dissipate the increasing heat flux becomes a central concern one that directly impacts reliability, longevity, and overall operational integrity [12,13,14].

Numerous studies and design strategies have been devoted to addressing the escalating thermal challenges inherent in advanced heterogeneous integration. As the stacking of multiple active layers in 3D-ICs and chiplet-based systems restricts natural heat dissipation, engineers and researchers have explored various approaches to facilitate thermal spreading and extraction. Among these, the introduction of thermal through-silicon vias (TTSVs) stands out as a prominent solution [15,16].

Although significant progress has been made in thermal management, recent literature consistently emphasizes that the core issue of heat accumulation remains largely unresolved. The use of through-silicon vias (TTSVs) [17,18], the introduction of high-conductivity materials [19], and the adoption of advanced multiphysics modeling have brought incremental improvements [20]. However, these measures are not sufficient to overcome the increasing complexity of stacked architectures and the continuous rise in power density [21,22]. As a result, research studies highlight the need for integrated and multidisciplinary strategies combining material innovations, architectural optimizations, dynamic thermal control techniques, and advanced cooling methods to effectively contain thermal effects, even in the most recent system generations.

### 1.3. Transition Toward Emerging Security Concerns: Thermal Side-Channel Attacks (TSCAs)

In the face of persistent thermal constraints in advanced microelectronic systems, a new and insidious threat has emerged: the malicious exploitation of thermal side effects through thermal side-channel attacks (TSCAs) [23]. Cyber attackers have now realized that temperature variations caused by internal circuit activity can unintentionally reveal sensitive information such as computation patterns, application workloads, or even protected engineering secrets. Far from being passive thermal byproducts, these emissions become exploitable information channels, enabling adversaries either local or remote to stealthily and non-invasively infer critical aspects of a system’s operation. This emerging yet rapidly evolving field highlights that thermal management is no longer solely a matter of performance or device reliability, it has become a crucial component of embedded system cybersecurity. As heterogeneous integration and functional density continue to grow, protection against TSCAs is becoming a strategic imperative to ensure the confidentiality, integrity, and robustness of next-generation hardware architectures [24].

### 1.4. System-Level Impact of Thermal Side-Channel Vulnerabilities

As advanced VLSI and ULSI chips form the computational core of cyber–physical systems (CPSs) and internet of things (IoT) infrastructures, ensuring the security of these microarchitectures has become a strategic imperative [25,26]. Far from being standalone components, these chips are integral to systems that orchestrate real-time data acquisition, processing, and actuation, serving as the essential bridge between computational logic and physical behavior. Consequently, any hardware-level vulnerability, particularly those involving thermally exploitable side channels such as TSCAs, can propagate beyond the chip itself, undermining the integrity of system-wide operations, from sensor actuator coordination to safety-critical control loops.

### 1.5. Paper Objectives and Specific Contributions

**Figure 2:**
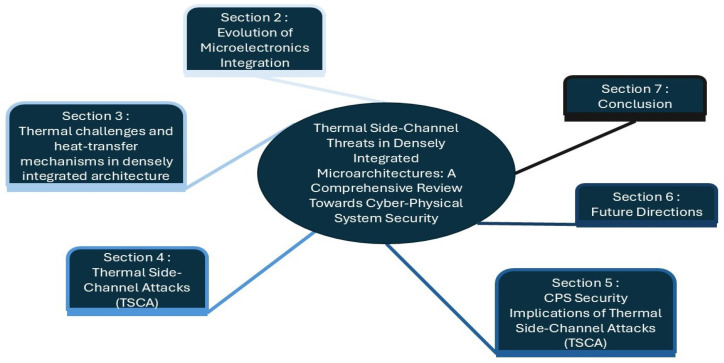
Mind map of the main sections covered in the TSCA-focused review article.

To provide a structured and comprehensive understanding of thermal side-channel threats in densely integrated microarchitectures, this review follows the conceptual structure illustrated in the mind map in Figure 2. Section 2 traces the evolution of microelectronics integration from monolithic systems to heterogeneous and 3D assemblies and sets the technical context. Section 3 characterizes heat-transfer mechanisms and thermal challenges specific to dense stacks and frames thermal side channels at a high level. Section 4 develops the thermal side-channel theme itself, formalizing definitions and mechanisms and surveying recent attacks across platforms. Section 5 analyzes the security implications for cyber–physical systems, positioning TSCA defenses within broader CPS trustworthiness and policy. Section 6 outlines future directions, with emphasis on digital twin modeling and AI-driven analysis for detection and mitigation. Section 7 concludes the paper.

## 2. Evolution of Microelectronics Integration: From One Chip to 3D and Heterogeneous Architectures

### 2.1. The Age of Monolithic (One-Chip) Integration

Initially, microelectronic integration centered on the monolithic “one-chip” paradigm, where all electronic functions, logic, memory, analog, and interface, were implemented on a single silicon die using the same technological process. This strategy allowed for relentless miniaturization, largely in step with Moore’s Law, which propelled increases in integration density and functionality while lowering cost per function [27]. However, as transistor scaling approached physical and economic limits at the nanometer scale, new challenges such as short-channel effects, device variability, and escalating fabrication complexity began to hinder continued progress [28].

In addition to these technical limitations, the planar approach encountered bottlenecks in performance due to longer interconnect distances, growing power density, and heat dissipation difficulties [29]. These factors compelled industry and academia to reimagine how integration could meet the growing needs of AI, IoT, automotive, and medical applications [30].

### 2.2. The Rise of Heterogeneous Integration

To overcome the constraints of monolithic integration, the semiconductor industry has progressively adopted heterogeneous integration. This involves assembling multiple components potentially built using different semiconductor technologies or materials into a unified system at the package or even die level [31]. The approach provides flexibility by allowing each functional block (logic, memory, sensors, photonic devices, MEMS, etc.) to be fabricated with the process best suited to its requirements.

Several forms of heterogeneous integration have emerged:

- System-in-Package (SiP): Multiple chips combined in a single package.
- Heterogeneous SoCs: Integration of diverse functions within a single die or closely coupled dies.
- Chiplets: Functional blocks separately manufactured and dynamically assembled on advanced interposers [32].

The increased flexibility enables system designers to use the right technology for the right function without being limited to a single process, boosting performance and power efficiency. It also improves manufacturability, speeding design cycles and making yield higher by localizing defects to small dies rather than massive monoliths [33].

### 2.3. Three-Dimensional Integration: Leveraging the Vertical Dimension

**Figure 3:**
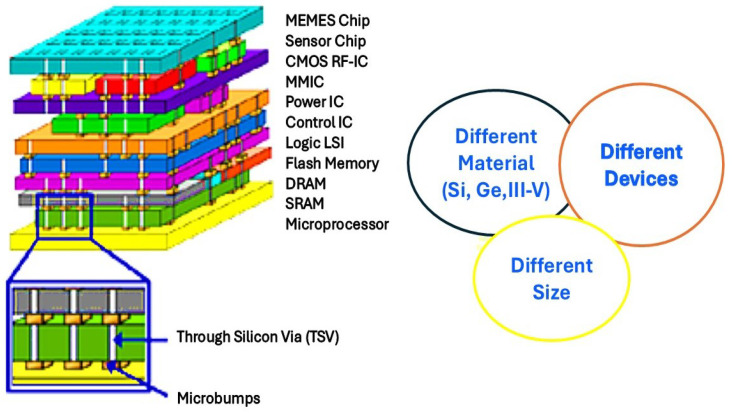
Heterogeneous 3D super-chip architecture integrating multifunctional device layers via TSVs and microbumps.

Moving beyond two-dimensional constraints, 3D integration employs stacking of chips or wafers (often with through-silicon vias (TSVs) and hybrid bonding) to further increase packing density and bandwidth while reducing power and latency. Vertical integration allows close proximity between different functional dies (such as memory on top of logic), minimizing wiring length and enabling new architectures impossible with 2D only designs [34]. A representative example is illustrated in Figure 3, which shows a super-chip architecture where diverse materials and technologies including CMOS, RF ICs, MEMS, power ICs, and sensors are vertically integrated via TSVs and microbumps. This configuration highlights the capability of 3D integration to support heterogeneous stacking involving different materials (e.g., Si, Ge, III–V), devices, and sizes within a compact volume. Three-dimensional stacking is especially attractive for high-performance applications, as it allows substantial increases in data bandwidth and system compactness, which are critical for edge computing, autonomous vehicles, and data centers [35]. Multifunctional stacking of logic, memory, photonics, MEMS, and sensors opens new device classes, from smart wearables to biomedical implants to AI accelerators [36].

### 2.4. Technical and Industrial Challenges in Microelectronics Integration: Long-Term Visions of Major Industry Players

The rapid evolution of microelectronics integration technologies, particularly in the realms of 3D heterogeneous stacking and chiplet modular assembly, has introduced unprecedented complexities on both technical and industrial fronts. As semiconductor devices increasingly incorporate multiple dies, heterogeneous materials, and intricate interconnects within compact packages, the challenges multiply in terms of design complexity, manufacturing precision, thermal management, and reliability assurance. These challenges are compounded by the economic pressures inherent to scaling production to volumes demanded by global markets [35]. Thermal management emerges as a particularly critical concern. The stacking of multiple active layers in vertical configurations raises the power density within limited volumes, aggravating hotspots and thermally induced stresses that directly impact device longevity and performance stability [37]. Concurrently, maintaining high yield and consistent quality across multi-die assemblies encounters manufacturing barriers resulting from the fragile interfaces, alignment constraints, and material mismatches inherent to these advanced integration schemes. These factors place significant pressure on fabrication lines to develop sophisticated inspection, bonding, and testing solutions tailored for heterogeneous systems [38]. Recognizing these hurdles, leading semiconductor companies such as Intel, TSMC, and Samsung have articulated and deployed long-term strategic visions that emphasize innovation in integration technologies, manufacturing processes, supply chain robustness, and ecosystem collaborations. These industry titans play crucial roles in setting technology roadmaps and funding R&D initiatives aimed at overcoming integration bottlenecks while enabling scalable and cost-effective production. This section explores these corporate strategies and technological pathways as responses to the multifaceted challenges in microelectronics integration.

Leading semiconductor companies have revolutionized electronic integration through specialized advanced packaging approaches that enable high-density, modular, and heterogeneous system design:

- Cadence Design Systems: Their Integrity 3D-IC platform developed in collaboration with TSMC provides a holistic toolchain for co-designing, verifying, and optimizing complex 3D and chiplet-based architectures. By combining floorplanning, power and thermal integrity analysis, and multiphysics simulations in a single environment, such platforms allow for early detection of thermal hotspots and design rule violations, ultimately accelerating design cycles and enhancing reliability [39,40]. Cadence’s strategic vision, reflected in its alliance with TSMC 3DFabric technologies and the integration of AI-enhanced EDA tools like Celsius and Voltus, exemplifies how industrial ecosystems are responding with innovation-driven methodologies to overcome current integration bottlenecks
- Intel introduced Embedded Multi-die Interconnect Bridge (EMIB) and Foveros. EMIB facilitates high-bandwidth chiplet integration without a full silicon interposer, while Foveros enables true 3D logic-on-logic stacking. These platforms support flexible, high-performance assemblies critical for AI and data center applications [41].
- AMD led mainstream adoption of chiplet-based CPUs and GPUs using Infinity Fabric for high-bandwidth, coherent interconnects. Its 3D V-Cache, using direct copper-to-copper bonding, exemplifies how packaging can drive performance gains and better yield through small die architectures [42].
- Apple and TSMC partnered on commercializing highly integrated 2.5D/3D packaging with System-on-Integrated-Chip (SoIC) and Integrated Fan-Out (InFO). InFO packaging enables ultra-dense, low-profile, wafer-level integration pivotal for compact, power-efficient mobile processors, while SoIC brings fine-pitch heterogenous die stacking to mainstream consumer electronics [43].
- NVIDIA advanced High-Bandwidth Memory (HBM) integration with Chip on Wafer on Substrate (CoWoS), which bridges GPU logic and memory through silicon interposers. This design achieves the massive bandwidth and efficiency required by AI and machine learning workloads [44].
- Samsung developed X-Cube and H-Cube packaging, focusing on vertical stacking of memory and logic with fine-pitch TSVs. These platforms enable lower interconnect delays and dense logic memory integration, supporting demands of mobile, server, and AI applications [45].

Each of these case studies demonstrates that advanced packaging has become central to overcoming the scaling, performance, and integration limits of conventional silicon designs. Industry-wide, collaborations among foundries, design houses, and assembly test partners fuel continued innovation and modularization of complex electronic systems.

### 2.5. Evolution of Integration Paradigms—A Summary and Insights

An analysis of Table 1 reveals a recurrent technical barrier that transcends all integration paradigms of thermal management. Whether in traditional monolithic SoCs or in cutting-edge 3D-stacked and heterogeneous architectures, the accumulation and dissipation of heat remain systemic challenges. In monolithic designs, increasing transistor density leads to elevated power densities and localized hotspots. With the evolution toward 3D stacking and chiplet integration, the vertical proximity of active dies exacerbates thermal coupling, making traditional heat spreaders and sinks insufficient.

## 3. Thermal Challenges and Heat-Transfer Mechanisms in Densely Integrated Architecture

**Figure 4:**
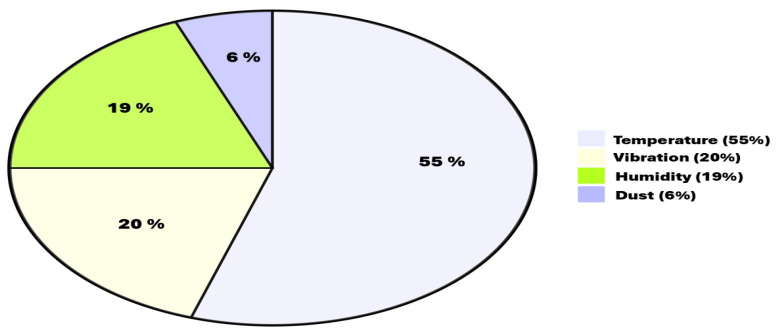
Electronic system failure causes [46].

The primary factors contributing to electronic failures are high temperature, humidity, vibration, and dust, as illustrated in Figure 4. Over half of all electronic system failures, roughly 55%, stem from inadequate thermal management [46]. The rise of dense integration methodologies such as 3D stacking and chiplet-based heterogeneous packaging has intensified thermal management issues. As power density increases and dies become vertically and laterally compacted, dissipating heat efficiently becomes a critical concern. This necessitates innovative strategies for thermal-aware design, material selection, package layout, and simulation techniques.

### 3.1. Heat-Transfer Mechanisms in Densely Integrated Architecture

In physics, three fundamental modes of heat transfer, conduction, convection, and radiation occur naturally within electronic equipment [47,48]. Below, each mechanism is described in the context of microelectronic packaging and printed-circuit-board (PCB) assemblies, together with the governing mathematical formulations.

#### 3.1.1. Conduction

Thermal conduction in electronics refers to the transfer of heat through a solid material or between solids in direct contact, such as from a semiconductor die through its package into the PCB. The local heat flux *q* (W/m^2^) is governed by Fourier’s law [48]:

(1) q=−k∇T

where

- *k* (W·m^−1^·K^−1^) is the thermal conductivity of the medium;
- ∇T (K·m^−1^) is the temperature gradient.

In one dimension, this reduces to

(2) $$q_{x}=-k\;\frac{dT}{dx}\;,$$

and integrating across a thickness *L* yields the total heat flow rate *Q* (W) through area *A*:

(3) $$Q=-k\;A\;\frac{T_{\mathrm{hot}}-T_{\mathrm{cold}}}{L}\;.$$

#### 3.1.2. Convection

Convection describes heat transfer between a solid surface and an adjacent fluid (liquid or gas) [49]. Two regimes are common:

1. Natural convection, where fluid motion arises from buoyancy forces due to temperature-dependent density differences.
2. Forced convection, where an external device (fan, pump) imposes fluid motion.

The convective heat-transfer rate is given by Newton’s law of cooling:

(4) $$Q_{\mathrm{conv}}=h\;A\;\left(T_{s}-T_{\infty }\right)$$

where

- *h* (W·m^−2^·K^−1^) is the convective heat-transfer coefficient;
- *A* (m^2^) is the wetted surface area;
- $T_{s}$ (K) is the solid-surface temperature;
- $T_{\infty }$ (K) is the bulk fluid temperature.

#### 3.1.3. Radiation

Thermal radiation is the emission of electromagnetic waves (primarily in the infrared range) by a hot surface. When two surfaces are within each other’s radiative view, the net heat exchange follows the Stefan–Boltzmann law:

(5) $$Q_{\mathrm{rad}}=\varepsilon \;\sigma \;A\;\left(T_{s}^{4}-T_{\mathrm{amb}}^{4}\right)$$

where

- ε is the surface emissivity (dimensionless);
- $\sigma =5.670\times 10^{-8}\;W\;m^{-2}\;K^{-4}$ is the Stefan–Boltzmann constant;
- $T_{s}$ and $T_{\mathrm{amb}}$ (K) are the temperatures of the surface and surrounding environment, respectively;
- *A* (m^2^) is the radiating area.

In microelectronics, infrared radiation (IR) is frequently neglected in thermal models of integrated circuits, particularly under forced convection conditions [50,51]. However, it is important to recognize that radiation plays a critical role in infrared thermography (IRT), where emitted IR energy provides valuable information on both temperature distributions and heat fluxes within electronic assemblies.

**Figure 5:**
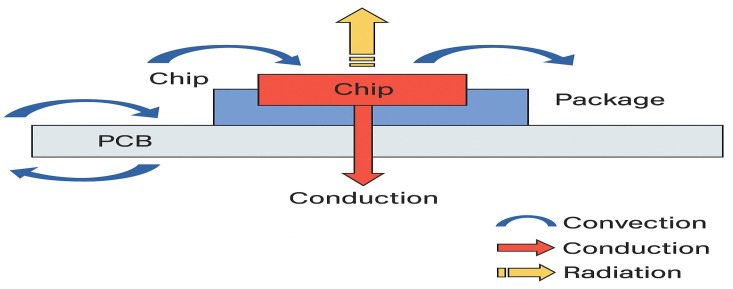
Heat-transfer pathways from the core of integrated architectures.

Figure 5 summarizes the three main mechanisms of heat dissipation in densely integrated architectures. These mechanisms must be considered collectively in any thermal modeling or cooling design of advanced electronic circuits.

### 3.2. Origin and Propagation of Thermal Stress in Monolithic and Heterogeneous IC Technologies

When an electric current flows through a conductor, heat is generated due to the inherent ohmic resistance present in all electrical conductors [52]. As a result, a portion of the electrical energy is converted into thermal energy, as described by Joule’s law (Equation (6)), which governs resistive (ohmic) heating.

(6) $$Q=I^{2}Rt$$

where

- *Q*: Thermal energy generated (in joules).
- *I*: Electric current flowing through the conductor (in amperes).
- *R*: Electrical resistance of the conductor (in ohms, Ω).
- *t*: Time during which the current flows (in seconds).

However, in all electrical and electronic components, the flow of electric current inevitably leads to heat generation. The power dissipated, $P_{D}$, expressed in joules per second [J/s] or watts [W], as shown in Equation (7), represents the portion of electrical energy converted into heat. This process is analogous to a flame fueled by the very electricity that sustains it.

(7) $$P_{D}=I^{2}R$$

**Figure 6:**
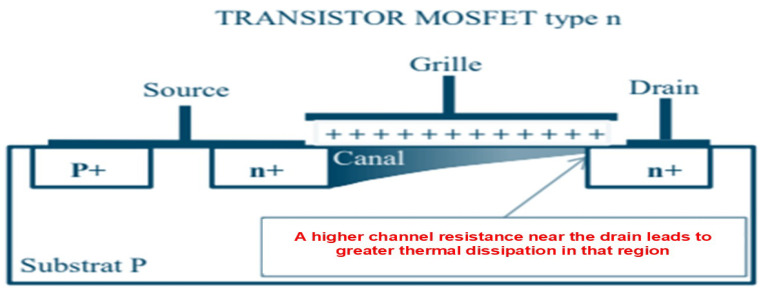
Heat dissipation profile in an n-MOSFET: increased resistance near the drain [48].

In CMOS-based technologies such as SoC, 2D-IC, and 3D-IC architectures, the field-effect transistor (FET), particularly the metal–oxide–semiconductor field-effect transistor (MOSFET), is inherently a source of thermal issues due to its operating principles and physical characteristics. When the transistor conducts, allowing current to flow through its channel, a certain amount of power is dissipated as defined by Equation (7). Figure 6 illustrates the internal structure of an n-channel MOSFET. The physical behavior of this device results in a non-uniform distribution of electric charge carriers (electrons) along the channel [53].

As a consequence, the electrical resistance is not constant, but rather depends on the spatial distribution of charge carriers within the channel. Most of the heat is therefore dissipated near the drain region, where the channel becomes narrower and the local resistance reaches its maximum.

Modern integrated circuits (ICs), including microprocessors, SoCs, and 2D/3D SiPs, contain billions of transistors densely packed within a limited silicon footprint. This leads to extremely high power densities, as large amounts of electrical energy are converted into heat over very small areas.

To illustrate the severity of this issue, consider the following analogy: A standard 60-watt incandescent light bulb, which radiates its heat over an approximate surface area of 120 cm^2^, becomes too hot to touch by hand. In contrast, a high-performance processor such as the Intel Core i9 dissipates a similar 60 W of power, but over a die area of only 3 cm^2^. This results in a heat flux nearly 40 times greater, highlighting the severity of thermal concentration in modern microelectronics. Despite being widely used in consumer laptops, the Core i9 and similar processors face critical thermal management challenges. The need to dissipate high power within a compact and thermally constrained environment such as a laptop chassis has become a major design constraint. Consequently, heat dissipation in such densely integrated circuits has emerged as a significant technological bottleneck.

### 3.3. From Device Hotspots to Chip/Package Heat Flow

The drain-proximal hotspot in a MOSFET is not an isolated phenomenon; in modern chips, billions of such devices act as distributed heat sources. To understand how these local sources become observable temperature signals, we must step up to the chip and package scales and examine the physical stack and its thermal pathways.

**Figure 7:**
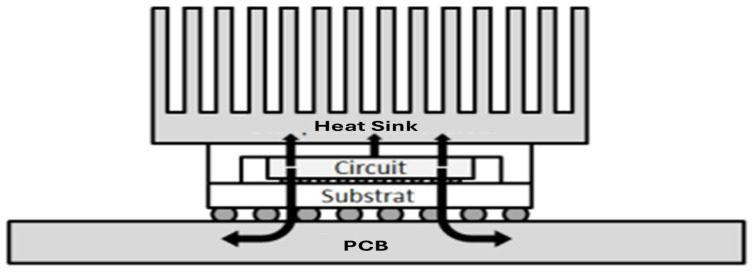
Typical structure of an integrated circuit [48].

An integrated circuit consists of a protective package that houses one or more semiconductor dies. The die, the functional core of the IC, is typically rectangular and contains the implanted circuitry. It is fabricated as a thin plate of semiconductor material, most commonly silicon, into which the transistors required for circuit operation are patterned. Lateral dimensions generally range from 1 to 3 cm, with a thickness of a few hundred micrometers. Due to manufacturing and structural constraints, the die size rarely exceeds ∼3 cm in either length or width. Figure 7 below illustrates the characteristic structure of a conventional integrated circuit.

A chip is fabricated on a crystalline silicon substrate onto which multiple material layers are sequentially built up to host the transistors and interconnects. Manufacturing proceeds in a layer-by-layer process (thin-film deposition, doping/oxidation, etching, planarization). The transistors and interconnect networks are defined by photolithography [54].

**Figure 8:**
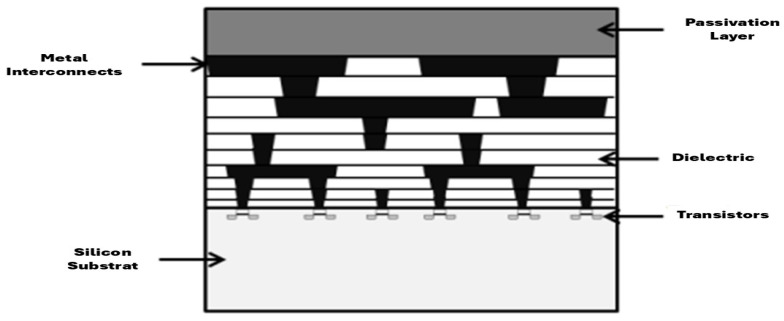
Composition of an integrated-circuit die [48].

Each new layer of the chip is meticulously coated with a photosensitive resist, which receives patterns projected by light filtered through a mask. The areas not protected by the resist are then carefully removed by a chemical process. This procedure is repeated for each layer of the chip, using its own set of masks. Finally, a crucial step envelops the circuit with a protective layer, a passivation layer composed of an oxide. Figure 8 depicts the superposition of layers that structures an IC chip.

In the study and finite element method (FEM) simulation conducted by Amrou Zyad Benelhaouare et al. [20], the results confirm the hypothesis that heat dissipation in modern integrated circuits is funneled through a small number of dominant pathways, upward to the encapsulation and ambient, laterally within the die/interconnect stack, and downward into the substrate whose relative contributions are set by the boundary conditions and stack materials. Consequently, device-level hotspots propagate along these paths and become observable at the chip and package scales.

**Figure 9:**
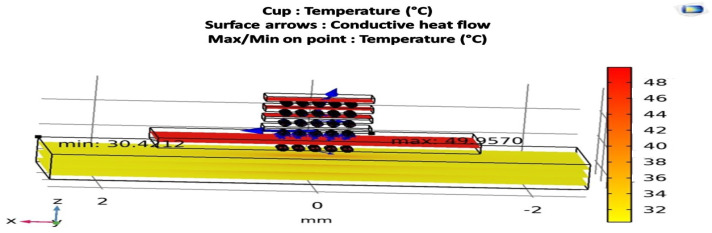
Heat-transfer pathway hypothesis [20].

Figure 9 presents a FE thermal simulation that visualizes the heat flux field with surface arrows. Three distinct routes emerge:

- An upward path from active regions through the interconnects to the passivation, package lid, and surrounding air;
- A lateral in-plane spreading within the silicon/metal layers toward neighboring blocks;
- A downward path into the silicon substrate and further to the PCB.

The simulation locates the hotspot on chip 1 localized in the middle with a peak temperature of ∼49.95 °C, consistent with the imposed boundary conditions, while the coolest region lies near the thermally anchored base at ∼30.42 °C. Overall, the vector field confirms the hypothesis: vertical extraction dominates under the chosen convection setting, whereas lateral spreading governs short-range thermal coupling between adjacent functional units.

### 3.4. Thermal Impedance Representation of Dense Microarchitectures

**Figure 10:**
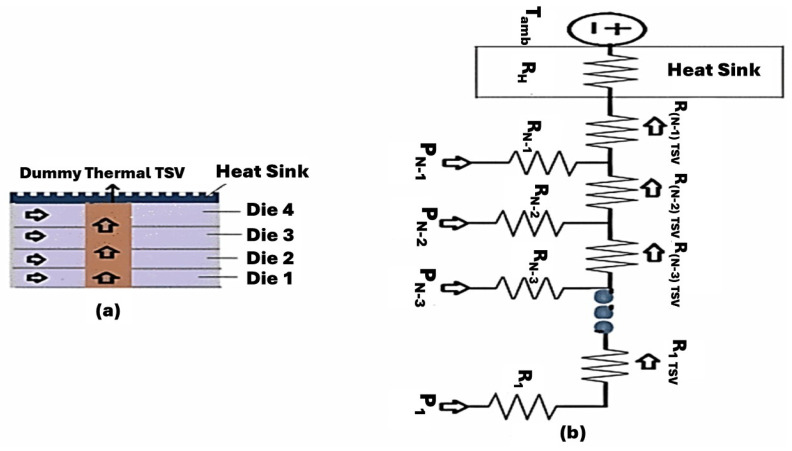
Vertical Heat Conduction via Thermal TSVs: (**a**) Physical 3D-IC Stack, (**b**) Lumped Thermal Network [55].

Building on the heat flow pathways confirmed in Figure 9, the 3D stack can be abstracted as a thermal impedance network in which each die, interface, and cooling element is mapped to a lumped element. In this heat–electrical analogy, heat flux plays the role of current and temperature rise the role of voltage. Vertical conduction through each die and interface is captured by series thermal resistances R1, R2, etc., while through-silicon vias (TSVs)/microbumps provide parallel cross-plane branches (an effective $R_{TSV}$ per tier). The package/heat-spreader/heat-sink path is terminated by a boundary element $R_{h}$ to the ambient $T_{amb}$ (Figure 10). For lateral coupling within and across tiers, transfer (cross) impedances $Z_{k}j$ are included alongside self-impedances $h_{kk}$, allowing the layer-k temperature rise to be written compactly as

(8) $$\Delta T_{k}=\sum_{j}Z_{kj}\;P_{j}$$

On the left (a), a 3D-IC with four dies shows a column of thermal TSVs linking the tiers to the top heat sink, arrows indicate vertical heat conduction toward the sink. On the right (b), the stack is reduced to a thermo-resistive ladder: each die dissipates power ($P_{i}$) and is associated with a thermal resistance ($R_{i}$), while the ($R_{TSV}$) branches provide parallel cross-plane paths upward. The network is terminated by the heat-sink resistance ($R_{H}$) to the ambient ($T_{amb}$). Taken together, Figure 10 illustrates how a densely integrated microarchitecture can be modeled.

### 3.5. Thermal Management Techniques for Densely Integrated Architectures

To better grasp the multifaceted nature of thermal management in densely integrated systems, we conducted a narrative synthesis of ten recent and high-impact studies published in IEEE, MDPI, and Elsevier. These works span from materials engineering to architectural modeling and system-level design trade-offs, offering unique insights into the persistent heat dissipation challenges and emerging mitigation strategies.

One particularly compelling example is the work by [56] on high-thermal-conductivity insulators, which exemplifies how passive material engineering can drastically shift the thermal profile of 3D ICs. This article introduces a compact thermal modeling framework to evaluate how interlayer dielectric (ILD) materials influence heat dissipation in 3D integrated circuits (3D-ICs). Simulation results reveal that replacing conventional low-conductivity dielectrics (e.g., SiO_2_, ∼1.4 W/m·K) with high-thermal-conductivity materials like aluminum nitride (AlN, ∼200 W/m·K) or hexagonal boron nitride (hBN, in-plane ∼390 W/m·K) can reduce peak temperatures by up to 15–20 °C in logic-on-logic configurations and over 25 °C in memory-on-logic stacks. Moreover, hBN’s strong thermal anisotropy enables directional heat spreading and up to 40% reduction in hotspot temperatures while maintaining thermal isolation between dies, a critical feature in heterogeneous architectures. These findings underscore that material-level substitutions alone can yield double-digit thermal improvements, positioning dielectric engineering as a powerful passive strategy for thermal optimization in next-generation 3D microelectronic systems.

Building upon the material-level strategies for passive heat conduction, recent advances have also explored layout-aware optimization as a thermal mitigation path. Notably, a 2023 study [57] introduced a multi-objective algorithm that simultaneously minimizes temperature and communication overhead in 2.5D IC architectures, leveraging chiplet placement flexibility to enhance both thermal and energy efficiency. This study proposes a novel multi-objective optimization algorithm tailored for 2.5D ICs with passive interposers, targeting simultaneous reductions in thermal buildup and communication overhead between chiplets. Inspired by Network-on-Chip (NoC) mapping techniques, the method introduces a tunable weight factor, α, enabling designers to balance temperature and communication energy according to system priorities. In experimental evaluations using a multi-window display system, the optimal configuration (with α=0.5) achieved a peak temperature reduction of 8.34K and communication energy savings of 232.13 μJ. Extreme tuning of α (e.g., 0.2 or 0.8) allows focused optimization toward either thermal performance or communication minimization, underscoring the algorithm’s adaptability. This approach exemplifies how layout-aware design and chiplet positioning can serve as an effective lever for passive thermal and energy management in 2.5D integration.

To address the intricate thermal and mechanical challenges in chiplet-based architectures, the study by [58] proposes a thermal–stress co-design methodology leveraging Support Vector Machine (SVM) modeling and the Particle Swarm Optimization algorithm with Linearly Decreasing Inertia Weight (PSO-LDIW). The method captures the complex and nonlinear relationship between the structural parameters of coaxial through-silicon vias (CTSVs) and key performance indices (temperature, stress in chip and copper columns). Remarkably, the optimized system achieved a peak temperature of 306.16 K and stresses of 28.48 MPa (chiplet) and 25.76 MPa (copper column), closely matching the desired targets (310 K, 30 MPa, 25 MPa). This machine learning-guided optimization significantly reduces design time (under 210 s per run), showcasing a practical and efficient route for the co-optimization of interconnect reliability and thermal integrity in advanced heterogeneous systems.

This study [59] tackles the growing challenge of thermal interaction in heterogeneous chiplet-based systems, particularly under high-performance computing and AI workloads. The authors propose two novel thermal modeling approaches, a collective parameter model and a distributed parameter model able to accurately predict thermal interactions between chiplets, validated against finite element method (FEM) simulations. Beyond modeling, the work introduces a thermal test vehicle to experimentally evaluate the effectiveness of different cooling solutions. Among them, liquid metal thermal interface materials (TIMs) show superior performance, reducing thermal coupling by 56% at 0.2 L/min and 76% at 0.8 L/min compared to conventional TIM (DOW5888). This enables chiplets to sustain heat fluxes up to 520 W/cm^2^ under optimized cooling, thus opening pathways for high-density, high-reliability integration in future chiplet architectures.

To further address the limitations of conventional simulation methods in advanced packaging environments, recent research has shifted focus toward in situ characterization tools. This article [60] introduces Thermal Test Chip (TTC) technology as a promising solution for capturing complex thermal and stress behaviors within microsystem interfaces during design and packaging processes. Unlike traditional finite element approaches, TTCs allow for direct in-package measurement, offering real-time insight into temperature gradients and mechanical stresses. The paper highlights how leading industry players such as Nanotest have developed TTCs based on JEDEC51-4A standards, contributing significantly to microsystem optimization. However, the current TTC implementations remain limited to single-parameter testing (e.g., temperature or stress). The article thus calls for the development of multiphysical field TTCs, capable of simultaneously monitoring stress, temperature, electromigration, and electromagnetic interference, addressing the increasing complexity and coupling of internal fields in chiplet-based and 3D-stacked microsystems.

To address the growing complexity of thermal analysis in chiplet-based 2.5D and 3D architectures, this work [61] presents a multi-fidelity thermal (MFIT) modeling framework that enables rapid and accurate temperature prediction across different abstraction levels. By combining finite element modeling (FEM), thermal RC networks, and discrete state space (DSS) models, MFIT provides scalable simulation solutions for both design time exploration and runtime control. Notably, the thermal RC models achieve 7× to 144× speedup over HotSpot, with a mean absolute error (MAE) below 1.7 °C, while the DSS models offer millisecond-scale predictions suitable for real-time thermal-aware scheduling. Across diverse workloads, including realistic AI and ML scenarios, MFIT consistently maintains temperature prediction errors under 1.6 °C and detects over 99% of thermal violations, validating its reliability and responsiveness in dense chiplet environments. The authors further provide an open-source implementation, making MFIT a powerful enabler for future research in dynamic thermal management and cyber–physical system (CPS) security.

In this new work [62], DeepOHeat proposes an ultra-fast, end-to-end thermal simulator for 3D-ICs based on operator learning, directly mapping configurations to temperature fields. DeepOHeat encodes, in a unified and modular fashion, stacked geometries, boundary conditions of various types (Dirichlet, Neumann, convection, adiabatic), 2D/3D power maps, and internal conductivity distributions as network inputs. Compared to the Celsius 3D FEM solver, DeepOHeat achieves a 1000× to 300,000× speedup on CPU and GPU, while maintaining high accuracy, with a Mean Absolute Percentage Error (MAPE) of 0.011% to 0.032% and Peak Absolute Percentage Error (PAPE) of 0.025% to 0.043%, with a maximum temperature deviation < 0.1 K.

These two studies [63,64] investigate complementary approaches for controlling thermal dissipation and mitigating mechanical stress in through-silicon vias (TSVs) within 3D-ICs. In the first study [63], the authors develop a global finite element analysis (FEA) model to accurately identify peak stress at the copper–memory interface of the via, demonstrating that these peaks arise primarily from mismatched coefficients of thermal expansion. A geometric sensitivity analysis reveals that reducing via diameter and increasing microbump thickness substantially lowers stress while preserving electrical conductivity. The second study [64] also employs FEA, applied to a 3×3 via array under an orthogonal experimental design, to quantify the effects of fill material (copper, aluminum, or tungsten), spacing, diameter, and height on thermal stress. The results indicate that fill material exerts the greatest influence, followed by spacing and diameter, with height having a comparatively minor effect.

Finally, the study [65] makes a key contribution by introducing a unified simulation framework that combines through-silicon thermal vias (TTSVs) and micro-heat sinks to simultaneously optimize passive and active thermal management in three-dimensional system-in-package (3D-SiP) assemblies. Using this approach, the network peak temperature falls from 70 °C to 45 °C (a decrease of 36 °C, i.e., 51%), while the SiP’s maximum internal temperature is reduced by 84% compared to previous methods.

This overview is by no means exhaustive; thermal management in densely integrated microelectronic systems remains an open challenge, and an expanding body of research continues to push the boundaries of what is possible.

**Figure 11:**
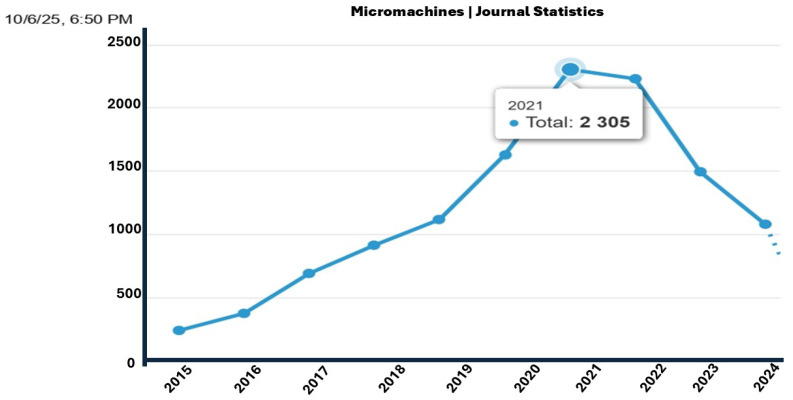
Annual Evolution of Publications Related to Thermal and Microelectronic Research (2015–2024) in MDPI’s Micromachines Journal.

Recent publication trends clearly demonstrate a growing interest in thermal related research within the microelectronics community. According to statistical data from the journal Micromachines (MDPI) [66], the number of papers published annually in this field has steadily increased between 2015 and 2022, with a peak observed in 2021 at nearly 2305 publications, before a slight decline in 2023 and 2024 (see Figure 11). This upward trend reflects a rising awareness of the importance of thermal phenomena in the reliability, efficiency, and security of advanced microarchitectures.

Moreover, among the top subjects covered in *Micromachines* between 2015 and 2025, domains directly related to thermal management and integration, such as microelectromechanical systems (1722 papers), semiconductor physics (1098 papers), and electronic packaging and interconnection (421 papers), stand out as active and evolving areas of research (see Table 2). These topics are closely intertwined with thermal behavior at the device and system levels, making them particularly relevant to the study of thermal side-channel vulnerabilities in densely integrated microarchitectures.

Such an increase in academic output confirms that thermal issues are no longer limited to power efficiency, but are now considered as potential security vectors in the design of secure and resilient cyber–physical systems (CPSs).

## 4. Thermal Side-Channel Attacks (TSCAs)

### 4.1. Background: Side-Channel Attacks (SCAs): Definitions, Taxonomy, and Scope

Side-channel attacks (SCAs) exploit statistical correlations between auxiliary physical effects and secret dependent computations. Instead of breaking the algorithm itself, an adversary observes one or more parasitic signals’ execution time, power draw, electromagnetic (EM) emanations, cache behavior, or temperature and infers sensitive information from their dependence on the computation [67,68,69,70,71].

A widely used taxonomy distinguishes the following:

- Passive channels: pure observation without perturbing the target (e.g., timing, power, EM, cache effects, thermal) [72,73,74].
- Active (fault-induced) channels: the attacker injects faults (e.g., clock/voltage glitches, overheating) to force incorrect behavior that reveals secrets [75].
- Covert channels: two principals communicate via a shared resource not intended for communication (e.g., shared core or shared thermal domain) [76,77,78].

Two additional axes help compare attacks:

- Degree of invasiveness: non-invasive (no opening/modification of the device), semi-invasive (limited exposure, e.g., delidding for closer sensing), invasive (direct silicon-level intervention) [79].
- Access vector: proximal hardware probing (contact or infrared (IR)), software-only observation (reading on-die (on-chip) sensors or thermal interrupts through the operating system (OS)), or co-resident processes on the same platform [69].

**Figure 12:**
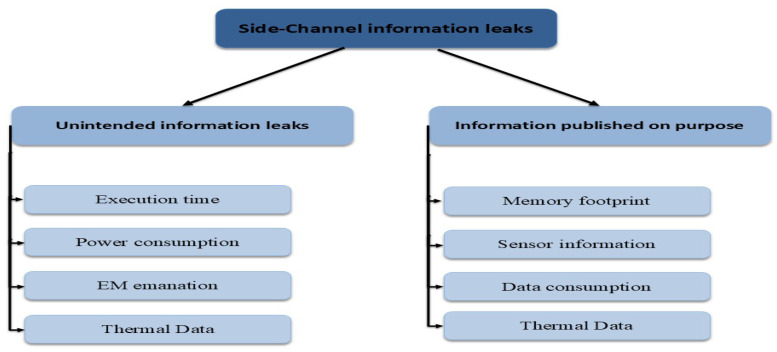
Side-channel information leaks.

The diagram in Figure 12 formalizes a taxonomy of side-channel information leaks according to the nature of the signal and the intention of exposure. The class of unintended leaks groups physical byproducts whose dependence on sensitive computation can be exploited, including execution time, power consumption, electromagnetic emissions, and thermal data when heat is measured by contact or infrared outside intended interfaces. The class of intentionally published information covers signals exposed for usability or monitoring, including memory footprint, sensor information, and data consumption, and it also includes thermal data when it comes from on-die sensors accessible through the OS. The presence of thermal data in both classes highlights a common physical mechanism: data-dependent power is converted into heat and filtered by the system’s thermal chain, which can be observed either as unintentional leakage or as instrumented telemetry, and this broadens the attack surface and shapes threat model assumptions and the choice of countermeasures.

### 4.2. The Mechanistic View of Thermal Side Channels (TSCs): From Observation Vector to Exploitation

#### 4.2.1. From the Observation Point to an Exploitable Signal

We begin with the observation vectors. They may be external, by contact sensing or infrared (IR) imaging; software-based, by reading temperature sensors integrated on die and exposed by the operating system (OS); or co-resident, when the attacker and victim share the same chip or package. In every case, the goal is to obtain a temperature trace that reflects, even if attenuated and delayed, the target’s computation activity.

#### 4.2.2. From Data-Dependent Activity to Power

The physical link starts at the complementary metal–oxide–semiconductor (CMOS) level: bit transitions cause capacitive charge and discharge. The dynamic power then follows:

(9) $$P_{\mathrm{dyn}}\left(t\right)\approx \alpha \left(t\right)\;C\;V^{2}\;f,$$

where α(t) captures switching activity, *C* is an effective switched capacitance, *V* is the supply voltage, and *f* is the clock frequency. For side-channel reasoning, simple predictors of activity such as Hamming Weight and Hamming Distance summarize, respectively, the number of ones in an intermediate value and the number of bit flips between successive states [67,80]. They are not an end in themselves, but a bridge from internal values to a tractable estimate of $P_{\mathrm{dyn}}\left(t\right)$.

#### 4.2.3. From Power to Temperature Along the Thermal Chain

The dissipated power becomes heat that propagates through a thermal chain comprising the silicon die, interfaces, lid, heat sink, and ambient air. Around an operating point, this propagation is efficiently modeled by a thermal impulse response $Z_{\mathrm{th}}$ so that the temperature rise is a convolution:

(10) $$\Delta T\left(t\right)\;=\;\left(Z_{\mathrm{th}}*p\right)\left(t\right).$$

This relation formalizes the intuition that the thermal path acts as a low-pass filter: it preserves slow variations of power and strongly smooths fast components.

When many blocks or many dies are present [81,82,83], the scalar impedance generalizes to a matrix, so the temperature at location *i* obeys

(11) $$\Delta T_{i}\left(t\right)\;=\;\sum_{j}\left(Z_{ij}*p_{j}\right)\left(t\right).$$

#### 4.2.4. From Physical Temperature to What the Attacker Sees

The measurement chain, sensor and readout electronics, adds its own dynamics and noise. The observed model becomes

(12) $$T_{\mathrm{meas}}\left(t\right)\;=\;\left(h_{s}*Z_{\mathrm{th}}\right)*p\left(t\right)\;+\;\eta \left(t\right),$$

where $h_{s}$ represents the combined response of the sensor and acquisition, and η(t) aggregates perturbations and quantization. This expression highlights two essential points for exploitation: the bandwidth is low, and the quality of temporal alignment between the victim’s activity and the observation is critical.

#### 4.2.5. From the Trace to Inference

Once the trace is collected, the attacker performs preprocessing to remove drifts and trends, selects windows where the activity is comparatively stationary, and then applies an estimator consistent with the physics. When the target algorithm is repetitive and well synchronized, a correlation between $T_{\mathrm{meas}}\left(t\right)$ and an activity predictor built from Hamming models often suffices, for example in a correlation power analysis adapted to the thermal bandwidth. When the target is more complex or noisier, learning-based approaches that use temporal and spatial features from multiple sensors become relevant.

#### 4.2.6. From Inference to Exploitation

Depending on the objective, exploitation typically takes three forms: first, key recovery by ranking hypotheses that maximize the correlation between predictor and trace; second, a covert channel: an emitting process modulates its load, and a co-resident receiving process reads temperature and decodes the modulation, usually at very low rate but with robust operation; third, faults and remanence, where one pushes temperature beyond specifications or exploits aging effects to induce revealing errors or to recover fragments of state.

#### 4.2.7. End-to-End Exploitation Chain of a Thermal Side-Channel Attack (TSCA)

**Figure 13:**
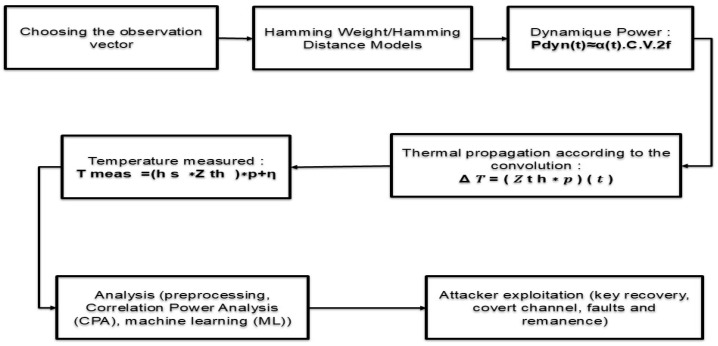
TSCAExploitation Pipeline (End-to-End).

The diagram in Figure 13 summarizes the full pathway from observation to exploitation. The adversary first selects the observation vector, performing external sensing with a contact probe or infrared imaging, software observation by reading on-die sensors exposed through the OS, or co-resident placement on the same chip or within the same package, and then links the targeted computation to a switching activity predictor such as Hamming Weight or Hamming Distance. This activity produces dynamic power as formalized in Equation (9); the resulting power waveform excites the device’s thermal resistance capacitance network and generates a temperature rise that follows the convolution model referenced in Equation (10). The sensor does not observe the physical junction temperature directly but a filtered and noisy signal, captured by the measurement model in Equation (12). Because the thermal transfer exhibits a low-pass spectrum, fast features are attenuated while slow variations are preserved, which motivates the use of windowing and alignment during preprocessing. The collected temperature traces are then processed by analysis methods, preprocessing, Correlation Power Analysis (CPA), and machine learning (ML), to infer secret dependent information. The attack culminates in key recovery, the establishment of a covert channel, or the exploitation of faults and remanence, and the effectiveness depends on bandwidth, spatial mixing, dynamic voltage and frequency scaling (DVFS), and the precision of sensor access.

Throughout this chain, several constraints determine practical success: the limited bandwidth imposed by the thermal transfer $Z_{\mathrm{th}}$ and the sensor response $h_{s}$, the spatial mixing introduced by thermal propagation paths, the closed loop between temperature and DVFS that injects nonstationarity, and the resolution, cadence, and access permissions of the temperature sensors. These factors are not merely impediments; they shape realistic threat models and steer the design of countermeasures.

Guided by these constraints, the next section organizes the literature by observation vector rather than by chronology, and evaluates each study under a uniform set of criteria: the method and the signal actually observed, the access vector (software via the operating system, external measurement, or co-resident processes), the platform under test, the primary measured outcome, and the practical strengths and limitations.

### 4.3. Recent Thermal Side-Channel Attacks (2015–2025): Observation Vectors, Platforms, and Outcomes

In this section, we survey recent thermal side-channel attacks from 2015 to 2025 with a focus on how the leakage is observed, where it is demonstrated, and what it achieves in practice. Rather than listing papers chronologically, we organize the material by observation vector: software access to on-die temperature telemetry through the operating system, co-resident scenarios on shared hardware such as multicores, GPUs, and cloud FPGAs, and external measurements using contact probes or infrared imaging. For each study, we apply the same evaluation lens: method and signal actually observed, access prerequisites and privileges, target platform and workload, primary outcomes such as data rate and accuracy, and a concise appraisal of strengths, limitations, and transferability. Throughout, we relate results to the physical constraints of the thermal path and sensor chain, including bandwidth, spatial mixing, dynamic voltage and frequency scaling, and measurement resolution, so that feasibility is judged within a realistic threat model.

#### 4.3.1. Software TSCAs on CPUs via Operating System Exposed Temperature Sensors and Thermal Events

To ground this framework, we begin with Masti et al. [84], the first comprehensive evidence that temperature telemetry on commodity multicore servers can support both a side channel and a covert channel. The authors read on-die digital temperature sensors exposed by the operating system, drive a heat source thread on one core, and recover the signal from a co-resident thread on another, achieving up to about 12.5 bits per second (bps) and detecting neighboring activity despite spatial and temporal partitioning. The contribution is compelling because it removes the need for external probes, but the channel is inherently low-bandwidth and depends on the availability, precision, and sampling cadence of OS temperature interfaces, which are policy-controlled and affected by dynamic voltage and frequency scaling. Building on this foundation, Bartolini et al. [85] developed an information-theoretic model for thermal covert channels in multicores, explicitly accounting for sensor quantization and noise. It proposes coding schemes and demonstrates rates above 45 bps on the same core and above 5 bps across one hop under favorable settings, thereby quantifying how thermal dynamics and sampling constraints bound capacity. The analysis crisply explains why careful coding helps reliability, yet it confirms the narrowband nature of thermal channels and their sensitivity to OS-level sensor policies.

Moving from external probes to software-only observation, this work [86] characterizes Intel’s digital thermal sensors and shows that user space access enables practical side channels. Turning to operating system exposed temperature telemetry, ThermalBleed demonstrates that on-die sensors can reveal microarchitectural activity to unprivileged code. With a focus on software interfaces, ThermalBleed shows that commodity temperature sensors provide enough signal to profile programs without elevated privileges.

Rather than polling temperature values, ThermalScope [87] exploits thermal event interrupts that the operating system raises when thresholds are crossed, including a browser-resident case with heat-amplifying techniques. This event-driven perspective improves observability and helps sidestep low sampling rates. Its practicality depends on how the OS configures thermal interrupts and on-sandbox policies, and the attack remains constrained by the underlying thermal bandwidth.

Under hardened power telemetry, Too Hot to Handle [88] shows that even when power telemetry is filtered to mitigate power side channels, adversaries can pivot to temperature and still extract high-level information such as browsing history in Chrome incognito mode and distinguish Tor traffic patterns. The paper forcefully argues that closing power interfaces is insufficient if temperature remains accessible, which shifts hardening priorities toward thermal sensor access control and rate or precision limiting. While details appear in preprint and proceedings venues, the message is clear and of immediate relevance to defenders.

#### 4.3.2. Co-Resident TSCAs on Densely Integrated Architectures and Accelerators

Extending TSCAs to cloud accelerators, Tian et al. [89] introduce a temporal covert channel in which one tenant’s heat residue is decoded by a subsequent tenant. The result establishes cross-tenant communication that requires no direct sharing, only temporal multiplexing of the same device. It convincingly extends the attack surface to cloud accelerators, but the achievable throughput is very low and depends strongly on environmental conditions and scheduler behavior.

Focusing on dense integration, Chen et al. [77] show that a system-on-chip (SoC) and its stacked Package-on-Package (PoP) DRAM can form a usable thermal covert link. By modulating load on the SoC and sensing at the DRAM side, the authors demonstrate inter-block signaling within a single package, highlighting how short thermal paths in modern packaging create cross-domain leakage. The scenario is realistic for mobile SoCs, though the channel quality is sensitive to workload scheduling noise, cooling policy, and sensor access privileges.

Shifting from CPUs to GPUs, González-Gómez et al. [90] demonstrate a practical thermal covert channel whose emitter is a GPU workload and whose receiver reads device telemetry such as temperature and frequency. The authors validate the channel on two platforms and argue that rates and error profiles are comparable to CPU-based channels, while discussing countermeasures adapted to GPU management. The result broadens TSCAs beyond CPUs, but feasibility hinges on driver exposure, power management modes, and access permissions to telemetry.

#### 4.3.3. Mobile TSCAs and Android Thermal Telemetry

At the mobile edge, Miedl et al. [91] show that standard Linux “thermal zones” on Android are sampled from user space and fed to sequence models that classify which applications are running over time, effectively turning routine thermal telemetry into a behavioral side channel. The study is important because it requires no external hardware and no elevated privileges, yet it also shows pronounced device and firmware specific variability, making portability and long-term robustness central challenges.

#### 4.3.4. TSCAs for Cryptography and Machine Learning

Targeting cryptographic workloads on multiprocessor systems on chip (MPSoCs), Aljuffri et al. [92] train convolutional neural networks to recover key-related information, then discuss extending thermal attacks to asymmetric cryptography and combining leakage with induced thermal faults. This demonstrates that machine learning can exploit the low-bandwidth but structured thermal signatures of cipher execution. The approach, however, requires profiling and large training sets, and generalization across DVFS policies and hardware variants remains an open issue.

Methodologically, Dey et al. [93] recast thermal time series as image-like inputs to convolutional networks and achieve robust classification across governor settings. The paper shows that CNNs can classify workload states and recover sensitive activity under different governor settings, underscoring that thermal leakage is amenable to data-driven inference. As with other learning-based approaches, the dependency on device-specific characteristics and training effort limits portability without additional adaptation.

Table 3 provides a compact overview of TSCAs reported between 2015 and 2025. For each study, it lists the observation vector actually used, the target platform, access prerequisites and privileges, the type and sampling cadence of the signal, the attack objective, and a very concise outcome summary. Terminology is harmonized to ease comparison across heterogeneous works, including software access to on-die temperature telemetry through the operating system, co-resident scenarios on shared hardware, and external measurements.

## 5. CPS Security Implications of Thermal Side-Channel Attacks (TSCAs)

### 5.1. Understanding CPS and the Rising Priority of CPS Security

Cyber–physical systems (CPSs) integrate digital, analog, physical, and human components within a single application, orchestrated through feedback loops in which physical processes influence computation and, in turn, are shaped by it [94,95]. This canonical view, widely adopted in academia, is codified by the National Institute of Standards and Technology (NIST) Framework for CPSs, which emphasizes the co-engineering of the cyber and the physical together with an overarching objective of trustworthiness that aggregates security, safety, privacy, reliability, and resilience [96,97]. Within this perspective, the NIST framework introduces a taxonomy formed by facet conceptualization, realization, and assurance across the CPS life cycle and by functional, business, human, trustworthiness, timing, data, composition, boundaries, and life cycle aspects, providing a common analysis lens to map threats and controls across layers, from real-time execution to human interaction.

**Figure 14:**
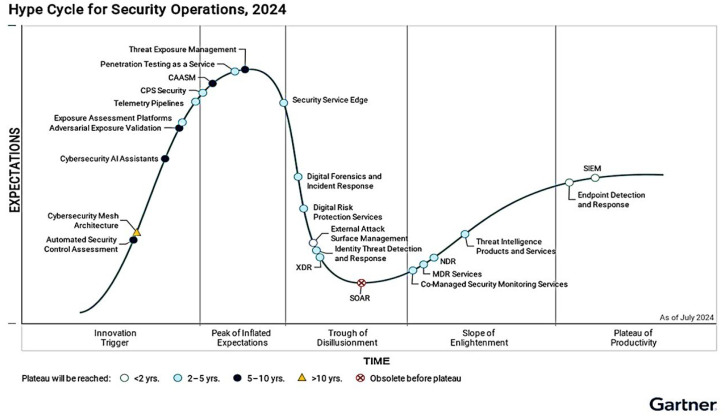
Hype Cycle for Security Operations, 2024.

In Gartner’s Hype Cycle for Security Operations 2024, CPS Security was positioned on the ascending slope, proximate to the Peak of Inflated Expectations, with a time-to-maturity estimated at approximately five to ten years (see Figure 14). This placement indicates high visibility and growing investment, alongside a gradually consolidating ecosystem of offerings; however, adoption remains heterogeneous, still characterized by pilots, proofs of concept, and governance frameworks that are maturing. In practice, this calls for phased roadmaps: (i) comprehensive CPS asset inventories, (ii) operational technology (OT)/information technology (IT) segmentation and zoning, (iii) dedicated operational monitoring, (iv) explicit sensor telemetry access policies (values and events), and (v) risk prioritization that explicitly accounts for physical consequences.

### 5.2. Nested Dependencies Between CPS Security and Thermal Side-Channel Defenses

**Figure 15:**
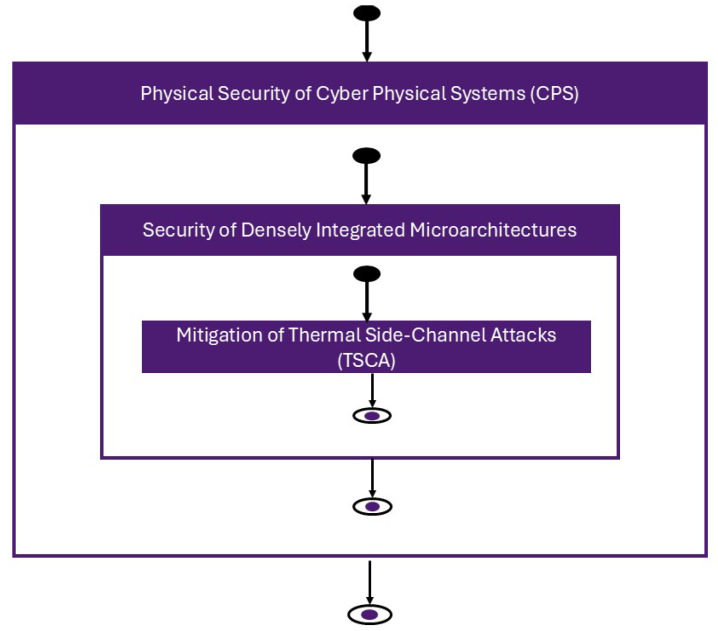
From CPS security to TSCA mitigation: a bidirectional dependency.

The diagram in Figure 15 conveys a nested yet bidirectional relationship between three levels of concern. At the outer level, the physical security of cyber–physical systems sets the system objectives confidentiality, integrity, availability, and safety and imposes operational constraints such as timing, energy limits, and maintainability. Inside this envelope sits the security of densely integrated microarchitectures, because VLSI/ULSI devices are the computational core that executes control logic and mediates sensor and actuator I/O. Nested again is the mitigation of thermal side-channel attacks, a specific but foundational facet of microarchitectural security: data-dependent activity generates power, power becomes heat, and heat can be observed or induced through on-die telemetry, co-resident workloads, or external probing. Strengthening TSCA defenses therefore hardens the microarchitecture and, by extension, the CPS.

The arrows emphasize that influence flows both ways. System-level requirements and safety policies shape the design of microarchitectures and their thermal interfaces, while microarchitectural vulnerabilities and protections fed back into the CPS pose risks by enabling or preventing leakage, covert signaling, and fault induction. In other words, TSCA mitigation is not an isolated patch, it is a lever that raises security one layer up, and CPS constraints, in turn, determine what mitigations are feasible and how they must be enforced.

This review is organized around that linkage. Read in light of Figure 15, the central message is straightforward: mitigating thermal side channels at the microarchitectural layer is a concrete and immediately actionable way to raise the physical security of CPSs as a whole.

### 5.3. Case Study—Industrial Control (PLC): Thermal Side-Channels and CPS Security

To strengthen the link between thermal side-channel attacks (TSCAs) and CPS security, we add a concise, real-world industrial example (Table 4). It shows how small, intentional temperature changes at the chip level can slow down control tasks, increase loop latency, and ultimately affect process safety.

### 5.4. Institutional Roles and Strategic Investments Relevant to CPS Security

#### 5.4.1. Department of Homeland Security (DHS)

Within the United States, the DHS through the Cybersecurity and Infrastructure Security Agency (CISA) and the Science & Technology Directorate supports the protection of CPSs across critical infrastructure by issuing guidance, coordinating public–private partnerships, and funding research and development focused on CPS/OT risk reduction. Recent CISA publications for operators of OT including asset inventory guidance, OT security principles, and procurement advisories illustrate sustained investment in practical controls such as OT/IT segmentation, visibility, and vulnerability management [98,99].

#### 5.4.2. National Science Foundation (NSF)

The NSF advances CPS security through competitive, peer-reviewed programs that fund foundational and use-inspired research in architecture, control, real-time and embedded systems, networks, safety and security co-design, and verification/assurance. The CPS program solicitations explicitly call for research that delivers trustworthy CPSs under adversarial and failure conditions and encourages validation via prototyping and testbeds, a clear signal of sustained federal investment in the CPS research ecosystem [100,101].

#### 5.4.3. Regional Artificial Intelligence Initiative and Cyber–Physical Resilience Program (RAII)

In Canada, the Regional Artificial Intelligence Initiative (RAII) provides substantial regional funding administered by the regional development agencies (RDAs). Under Budget 2024, PrairiesCan alone received approximately CAD 33.8 million over five years to deliver RAII (part of a broader national envelope of CAD 200 million for AI-powered businesses) [102]. These awards back projects that translate AI into production across priority sectors (e.g., manufacturing, clean technology, healthcare), enabling collaborations that can bridge advanced microelectronic design and next-generation cybersecurity in CPS contexts (for example, AI-driven methods for secure operation and monitoring on embedded platforms). Recent regional announcements highlight multi-million-dollar awards to institutes to accelerate adoption and commercialization, evidencing active investment at the CPS-relevant interface of AI, embedded systems, and security.

#### 5.4.4. Relevance to This Review

Taken together, these efforts delineate complementary responsibilities and investments: policy and operational coordination with practitioner guidance (DHS/CISA), foundational and translational research funding (NSF), and regionally targeted support to accelerate adoption in sectors where CPSs are mission-critical (RAII). By surveying and systematizing defenses against thermal side-channel attacks at the microarchitectural layer, this review addresses a concrete enabling lever for CPS security aligned with those strategic priorities.

## 6. Future Directions

### Open Research Directions: Digital Twins and Artificial Intelligence

**Figure 16:**
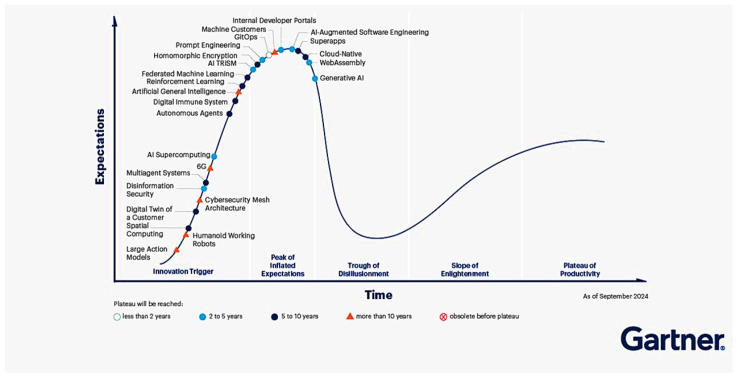
Hype Cycle for Emerging Technologies, 2024.

The two technology levers we highlight, digital twins (DT) and artificial intelligence (AI) (including machine learning, deep learning), are well positioned in Gartner’s 2024 Hype Cycles. As illustrated in Figure 16, the digital twin families appear in the early, high-traction zone (ascending slope toward the Peak of Inflated Expectations, with a time to plateau of 2–5 or 5–10 years depending on the variant), whereas core AI bricks (e.g., generative AI, AI-augmented software engineering) cluster near the peak with rapid adoption underway. This dual placement signals a maturing ecosystem and motivates targeted investment for adopting both technologies.

**Figure 17:**
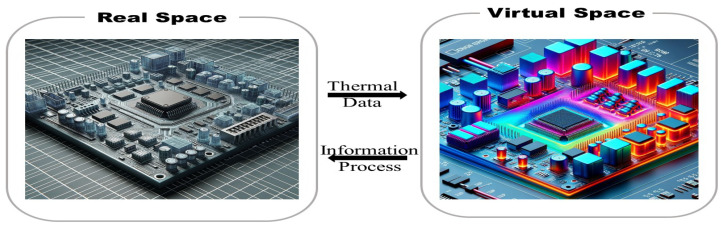
Thermal Digital Twin Concept.

Building on this observation, we propose combining these two concepts for thermal side-channel attacks (TSCAs) on densely integrated microarchitectures. As suggested by the real space ↔ virtual space schematic in Figure 17, thermal data (sensor values, thermal events, DVFS traces) are acquired in real time in the physical system, replayed and simulated within a twin of the thermal chain, and then analyzed by AI models capable of detecting weak spatiotemporal signatures: low-rate thermal modulation (covert channel), anomalous activity fingerprints, or indicators of fault induction.

Prior work [24,103] has shown that it is feasible to track temperature in real time, align traces with power models, and train classifiers to detect sensitive behaviors; the novelty here is to orchestrate these elements jointly so as to predict and flag TSCAs early despite the low bandwidth and spatial mixing intrinsic to the thermal path.

**Figure 18:**
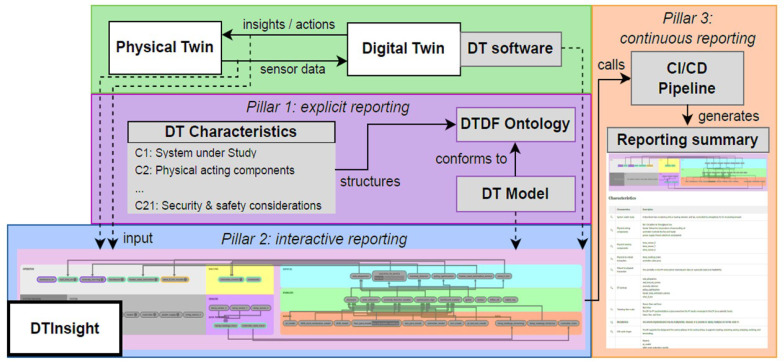
A Three-Pillar Digital Twin Framework for TSCA Defense [104].

We tentatively propose the following approach as a promising solution: DTInsight monitors the communication between the physical and the digital twin (DT), reads the modeled DT, and orchestrates TSCA analysis in a traceable and reproducible framework. Concretely, Figure 18 structures our approach into three pillars that, when applied to thermal leakage, offer a realistic path toward fully secured cyber–physical systems (CPSs):

Pillar 1—Explicit reporting:

We explicitly describe the system in the digital twin characteristics (available sensors, placements, access policies, boundary conditions) and link these elements to the digital twin description framework (DTDF) to form a DT model (layered, compact thermal macromodel tailored to 3D-IC/SiP/chiplets).

Pillar 2—Interactive reporting:

DTInsight aggregates streams (on-die/board/IR temperatures, dynamic voltage and frequency scaling (DVFS), power), executes the DT model to predict nominal behavior, then visualizes residuals (measured minus predicted). Artificial intelligence (AI) pipelines designed for sampled and quantized streams highlight low-frequency modulations characteristic of thermal covert channels.

Pillar 3—Continuous reporting:

A Continuous Integration/Continuous Delivery (CI/CD) pipeline rebuilds the twin on every change (code, model, data), replays standardized scenarios, and publishes a reporting summary (architecture snapshot, DT characteristics, metrics, provenance).

From a CPS perspective, adopting this approach will strengthen early detection of thermal leakages, reduce the attack surface, and improve operational resilience against adversaries exploiting the thermal chain.

## 7. Conclusions

This survey has shown that in densely integrated microarchitectures, including three-dimensional integrated circuits (3D-ICs), chiplet-based designs, and system-in-package (SiP) assemblies, heat is not merely a reliability concern but a security vector. We provided a unifying, physics-aware lens that connects data-dependent activity to power, power to temperature along the low-pass thermal chain, and temperature to attacker inference, and we organized the literature on thermal side-channel attacks (TSCAs) by observation vector. We related these threats to the security posture of cyber–physical systems (CPSs) and distilled cross-layer guidance on governance of sensor access and thermal events, thermal–DVFS co-design, sensor isolation and placement, controlled desynchronization and noise, and reproducible evaluation on real platforms.

Looking forward, we advocate the two complementary levers proposed in this work: thermal digital twins combined with artificial intelligence (AI) for early detection and prediction of TSCAs under bandwidth and spatial mixing constraints. Priority items include (i) open datasets coupling power, temperature, and system policies, (ii) compact thermal macromodels that scale to 3D-IC/SiP/chiplets, (iii) AI pipelines robust to sensor cadence and quantization with principled uncertainty handling, and (iv) metrics that balance detection latency, false alarms, and energy overhead. These directions align with current policy and funding momentum (e.g., DHS/CISA initiatives on CPS/OT security, NSF CPS programs, and Canada’s Regional Artificial Intelligence Initiative (RAII) Program), and they offer a practical route to harden microarchitectures and, by extension, to strengthen CPS trustworthiness.

## Figures and Tables

**Table 1:** Summary table: Evolutionary steps in integration.

Integration Paradigm	Description	Key Benifits	Main Challenges
Monolithic SoC	All functions on single silicon die	Mature, cost-effective, high-yield	Scaling, heat, flexibility
Heterogeneous Integration	Different device/processes combined in one system	Optimized functions, design modularity, flexibility	Fabrication, cost, thermal
3D-Stacked Integration	Multiple dies/layers stacked, interconnected vertically	Ultra-high density, reduced latency, BW boost	Heat management, complexity
Chiplets	Modular small dies integrated via interposer	Reusability, scalability, improved yield	Interface standardization, Heat
2D/3D Hybrid Materials	Atomically thin layers on 3D platforms	New electronic/optical properties, miniaturization	Reliability, integration, heat dissipation

**Table 2:** Top research subjects published in Micromachines (2015–2025), highlighting areas relevant to thermal management in densely integrated microarchitectures.

Subject	Publications
Microfluidic Devices & Surface Engineering	2029
Microelectromechanical Systems	1722
Semiconductor Physics	1098
Manufacturing	654
Wireless Technology	577
Organic Semiconductors	461
Electronic Packaging and Interconnection	421
Biosensors	402
Electrical Energy Management	386
Optoelectronics & Optical Engineering	373

**Table 3:** Recent TSCA studies (2015–2025): observation vector, platform, objective, and key outcome.

Year	Ref	Platform	Observation Vector	Access	Signal/Cadence	Objective	Outcome and Caveats
2015	Masti et al. [84]	×86 multicore CPUs	Software, co-resident	User OS	On-die temperature, Hz–tens of Hz	Covert, side	Low-rate covert side
2016	Bartolini et al. [85]	Multicore CPU	Software, co-resident	User OS	Quantized temperature	Covert	Capacity, coding gains
2019	Tian et al. [89]	Cloud FPGA	Co-resident (temporal)	Cloud tenant	Fabric temperature	Covert inter-tenant	Inter-tenant temporal channel
2019	Chen et al. [77]	SoC with PoP DRAM	Co-resident, cross-block	User OS	SoC/DRAM thermal coupling	Covert	Intra-package thermal coupling
2021	Aljuffri et al. [92]	MPSoC	Software/embedded	Varies	Windowed thermal traces	Crypto via ML	Crypto leakage ML
2021	Miedl et al. [91]	Android smartphone	Software	User OS	Linux thermal zones	Fingerprinting	App fingerprinting mobile
2021	Dey et al. [93]	Mobile edge MPSoC	Software	User OS	Thermal series to CNN	ML leakage	Workload classification ML
2023	González-Gómez et al. [90]	embedded GPU	Co-resident	User OS/driver	Temperature/frequency telemetry	Covert	GPU-based covert channel
2024	Zhang et al. [87]	x86 client	Software (event-driven)	User OS	Thermal interrupts	Side/covert	Event-driven side/covert
2024	Mishra et al. [88]	Intel CPUs (power defenses present)	Software	User OS	Thermal telemetry	Fingerprinting	Bypass power defenses

**Table 4:** Industrial Control (PLC): Thermal Side-Channel Impact on CPS Security.

Element	Content
System	Industrial controller (PLC) with a SoC/FPGA regulating a thermal process (e.g., an oven).
Exposed surface	On-chip temperature sensors, power/frequency logs, and automatic frequency scaling (DVFS).
Attack vector	Directed heating (e.g., IR/lamps) or crafted software load that raises local temperature.
Observable signal	Measured temperature changes and heat-driven shifts in frequency/power.
Technical effect	Processor/GPU throttling; control tasks execute later than planned.
Safety impact	Higher control-loop latency → setpoints may be overshot; triggers unsafe or unplanned shutdowns.
Reliability impact	Repeated thermal cycling accelerates component wear.
Practical mitigations	Restrict access to thermal/telemetry data; thermally isolate critical zones, alarms on fast temperature rise (rate-of-change), watchdogs that monitor control-loop latency.

## Data Availability

Data are contained within the article.

## Abbreviations

The following abbreviations are used in this manuscript:

3D-IC	Three-Dimensional Integrated Circuit
AI	Artificial Intelligence
CNN	Convolutional Neural Network
CMOS	Complementary Metal–Oxide–Semiconductor
CPA	Correlation Power Analysis
CPS	Cyber–Physical System(s)
CPU	Central Processing Unit
CISA	Cybersecurity and Infrastructure Security Agency
DHS	Department of Homeland Security
DRAM	Dynamic Random-Access Memory
DT	Digital Twin
DTDF	Digital Twin Description Framework
DVFS	Dynamic Voltage and Frequency Scaling
EM	Electromagnetic
FPGA	Field-Programmable Gate Array
FET	Field-Effect Transistor
GPU	Graphics Processing Unit
IC	Integrated Circuit
IR	Infrared
IRT	Infrared Thermography
IT	Information Technology
JEDEC	Joint Electron Device Engineering Council
ML	Machine Learning
MPSoC	Multiprocessor System-on-Chip
NSF	National Science Foundation
OS	Operating System
OT	Operational Technology
PoP	Package-on-Package
RAII	Regional Artificial Intelligence Initiative program
RC	Resistance-Capacitance (thermal RC network)
SCA	Side-Channel Attack
SiP	System-in-Package
SoC	System-on-Chip
TSCA	Thermal Side-Channel Attack
TSV	Through-Silicon Via
TTSV	Thermal Through-Silicon Via
